# Transient Global Amnesia Deteriorates the Network Efficiency of the Theta Band

**DOI:** 10.1371/journal.pone.0164884

**Published:** 2016-10-14

**Authors:** Young Ho Park, Jeong-Youn Kim, SangHak Yi, Jae-Sung Lim, Jae-Won Jang, Chang-Hwan Im, SangYun Kim

**Affiliations:** 1 Department of Neurology, Seoul National University College of Medicine and Clinical Neuroscience Center, Seoul National University Bundang Hospital, Seongnam, Korea; 2 Department of Biomedical Engineering, Hanyang University, Seoul, Korea; 3 Department of Neurology, Seoul National University Boramae Hospital, Seoul, Korea; 4 Department of Neurology, Kangwon National University Hospital, Chuncheon, Korea; Universidad de Jaen, SPAIN

## Abstract

Acute perturbation of the hippocampus, one of the connector hubs in the brain, is a key step in the pathophysiological cascade of transient global amnesia (TGA). We tested the hypothesis that network efficiency, meaning the efficiency of information exchange over a network, is impaired during the acute stage of TGA. Graph theoretical analysis was applied to resting-state EEG data collected from 21 patients with TGA. The EEG data were obtained twice, once during the acute stage (< 24 hours after symptom onset) and once during the resolved stage (> 2 months after symptom onset) of TGA. Characteristic path lengths and clustering coefficients of functional networks constructed using phase-locking values were computed and normalized as a function of the degree in the delta, theta, alpha, beta 1, beta 2 and gamma frequency bands of the EEG. We investigated whether the normalized characteristic path length (nCPL) and normalized clustering coefficients (nCC) differed significantly between the acute and resolved stages of TGA at each frequency band using the Wilcoxon signed-rank test. For networks where the nCPL or nCC differed significantly between the two stages, we also evaluated changes in the connections of the brain networks. During the acute stage of TGA, the nCPL of the theta band networks with mean degrees of 8, 8.5, 9 and 9.5 significantly increased (*P* < 0.05). During the acute stage, the lost edges for these networks were mostly found between the anterior (frontal and anterior temporal) and posterior (parieto-occipital and posterior temporal) brain regions, whereas newly developed edges were primarily found between the left and right frontotemporal regions. The nCC of the theta band with a mean degree of 5.5 significantly decreased during the acute stage (*P* < 0.05). Our results indicate that TGA deteriorates the network efficiency of the theta frequency band. This effect might be related to the desynchronization between the anterior and posterior brain areas.

## Introduction

Transient global amnesia (TGA) is an interesting syndrome of unknown etiology that is characterized by the abrupt onset of repetitive questioning. Despite the presence of profound amnesia, patients with TGA appear to have intact general cognition during the attack. They remain alert and communicative without focal neurological signs [1], and their memory usually starts to recover after a few hours, returning to normal within a day [2]. Emotional stress, physical efforts, temperature change and sexual intercourse are common precipitating events of TGA [2].

Because focal hyperintense diffusion-weighted imaging (DWI) lesions in area CA1 of the hippocampus have been reported during the acute stage of TGA [3], hippocampal CA1 injury and the subsequent perturbation of corticohippocampal circuits have been regarded as key steps in the pathophysiological cascade of TGA [4]. Before the documentation of these hippocampal DWI lesions, mesiotemporal hypoperfusion with concomitant involvement of various cortical and subcortical structures had also been noted in patients with TGA, suggesting alterations of the hippocampus and corticohippocampal circuits [5]. Recently, corticohippocampal disruption was identified within the episodic memory network during a TGA attack in a resting-state functional MRI study [6].

Graph theory enables the quantitative measurement of cortical connection features [7]. Path length, which is the number of edges that must be traversed to go from one node to any other node, is a key graph-theoretical index [8]. The average path length over all possible pairs of nodes, called the characteristic path length (CPL), is inversely related to the efficiency of information exchange over a network [8,9]. Given that the hippocampus is one of the major connector hubs in the brain [10], the perturbation of the hippocampal network during TGA might deteriorate the network efficiency.

In the current study, patients with TGA underwent resting-state EEG twice: once during the acute stage and once during the resolved stage of TGA. We investigated the hypothesis that acute TGA impairs network efficiency by comparing the CPL of the acute stage with that of the resolved stage in each frequency band. To examine whether another network characteristics are also affected during TGA, we evaluated the clustering coefficient (CC), which is related to the functional specificity of regional brain areas [11].

## Materials and Methods

### Participants

A retrospective analysis of patients with TGA was performed based on a registry database. We identified 22 patients who visited Seoul National University Bundang Hospital within 24 hours after symptom onset, between January 2008 and April 2014, and who fulfilled the TGA criteria [1]. The diagnostic criteria were as follows: (a) presence of anterograde amnesia that was witnessed by an observer, (b) absence of clouding of consciousness and loss of personal identity, (c) cognitive impairment limited to amnesia, (d) absence of focal neurological signs and epileptic features, (e) absence of recent history of head trauma and seizures, and (f) resolution of symptoms within 24 hours. The patients had 1- to 5-mm punctate hyperintense lesions in the lateral hippocampus on DWI (S1 Fig) [3]. Single-shot spin-echo echo-planar imaging was used for DWI with the following parameters: matrix, 128 × 128 interpolated to 256 × 256; field of view, 220 mm; repetition time, 9400 ms for 1.5 T (Intera; Philips Medical Systems, Best, Netherlands) and 5000 ms for 3 T (Intera Achieva; Philips, Best, Netherlands); echo time, 66 ms for 1.5 T and 59 ms for 3 T; SENSE factor, 2; number of acquisitions, 4; *b* value, 2000 s/mm^2^; and section thickness, 3 mm [12]. DWI was performed again at day 3 post-onset with the same parameters. Sixteen patients had hippocampal lesions on the initial DWI, whereas 6 patients had hippocampal lesions only on the follow-up DWI. The patients underwent EEG twice, once during the acute stage (< 24 hours after symptom onset) and once during the resolved stage (> 2 months after symptom onset) of TGA. After 1 patient was excluded due to EEG data that were unsuitable for analysis because of artifacts, the remaining 21 patients comprised the study population.

### EEG recordings

Spontaneous EEG was recorded in a resting state with the eyes closed. EEG data were acquired for 15 minutes from 21 electrode locations (Fp1, Fp2, F3, F4, F7, F8, Fz, C3, C4, Cz, T1, T2, T3, T4, T5, T6, P3, P4, Pz, O1, and O2) according to the international 10–20 system with a linked ear reference using a computer-based system (Natus Neurology, Inc, Warwick, RI). The EEG was recorded at a sampling rate of 200 Hz. The band-pass filter was set at 1 and 70 Hz, and a notch filter removed 60-Hz noise. The EEG data were visually inspected to obtain 20 series of 2-s epochs for analysis (400 samples) that were free of artifacts [13,14]. The data were then set to an average reference. The direct current offset component was subtracted in each epoch, and epochs exceeding ±75 μV in amplitude at any electrode were rejected from the analysis.

### Computation of the phase-locking value

Using 20 artifact-free epochs, the phase-locking value (PLV) was computed between all possible pairwise combinations of EEG electrodes. The PLV is a measure that quantifies phase synchronization between two signals that originate from different electrode locations but that were recorded during the same time interval and within the same frequency band [15]. The phases of two signals may be synchronized even when their amplitudes are not correlated [16]. The PLV is stationarity-independent and is focused on the phase of the signals. The PLV can range from 0 to 1. If the value is close to 1, the two signals are synchronized with a constant time lag. If it is close to 0, the two signals are temporally independent of each other. We made a 21×21 connectivity matrix for each of the following six frequency bands: delta (1.0–3.8 Hz), theta (4.0–7.8 Hz), alpha (8–12 Hz), beta 1 (12–18 Hz), beta 2 (18–26 Hz) and gamma (27–55 Hz) [17].

### Computation of the normalized CPL and normalized CC

The next step was to convert the weighted connectivity matrix into a binary adjacency matrix using a threshold. We constructed 999 unweighted binary networks by increasing the threshold from 0.001 to 0.999 with a step size of 0.001. The CPL and CC were evaluated for each binary network. The CC can be defined as the ratio of the number of existing connections between neighboring nodes and the maximum possible number of connections between neighboring nodes [9]. The local CC was first calculated for each node, and then the local CCs were averaged over all possible nodes in the network [18].

After calculating the two measures, the CPL and CC values were normalized using the corresponding values for 50 random networks [19] that were the same size but had different structures [20]. These random networks were generated by randomly changing the locations of the edges of the original network. Because graph theoretical measures are not only influenced by network structure but also by overall network size [21], we computed the normalized characteristic path length (nCPL) and the normalized clustering coefficient (nCC) as a function of the degree, which is the average number of edges per node [22].

### Statistical analysis

We compared the nCPL and nCC between the acute and resolved stage of TGA for networks across the six frequency bands using the Wilcoxon signed-rank test (*P* < 0.05). Networks for the acute and resolved stages that were equal in mean degree, which ranged from 5 to 10 in increments of 0.5, were compared [23]. We also calculated the effect size using Cohen’s *d* values [24]. Then, we evaluated the edges that were lost or developed in the brain network during acute TGA when the nCPL or nCC significantly differed. We also compared Bonacich centrality [25] between the acute and resolved stage of TGA for these networks using the Wilcoxon signed-rank test (*P* < 0.05). In addition, we compared the nCPL and nCC between patients with acute TGA and 44 age- and sex-matched control subjects using the Wilcoxon signed-rank test (*P* < 0.05). The control subjects were free of any neurological dysfunctions based on the 29 items of the Health Screening Exclusion Criteria [26] and had normal brain MRI examinations. Most statistical analyses were performed using STATA/SE version 14.0 (StataCorp, College Station, TX), while MATLAB 2015b (MathWorks, Inc, Natick, MA) was used for the EEG analysis. The study protocol was approved by Seoul National University Bundang Hospital institutional review board. Informed consent was waived due to the study’s retrospective nature and the minimal risk to participants.

## Results

**Fig 1:**
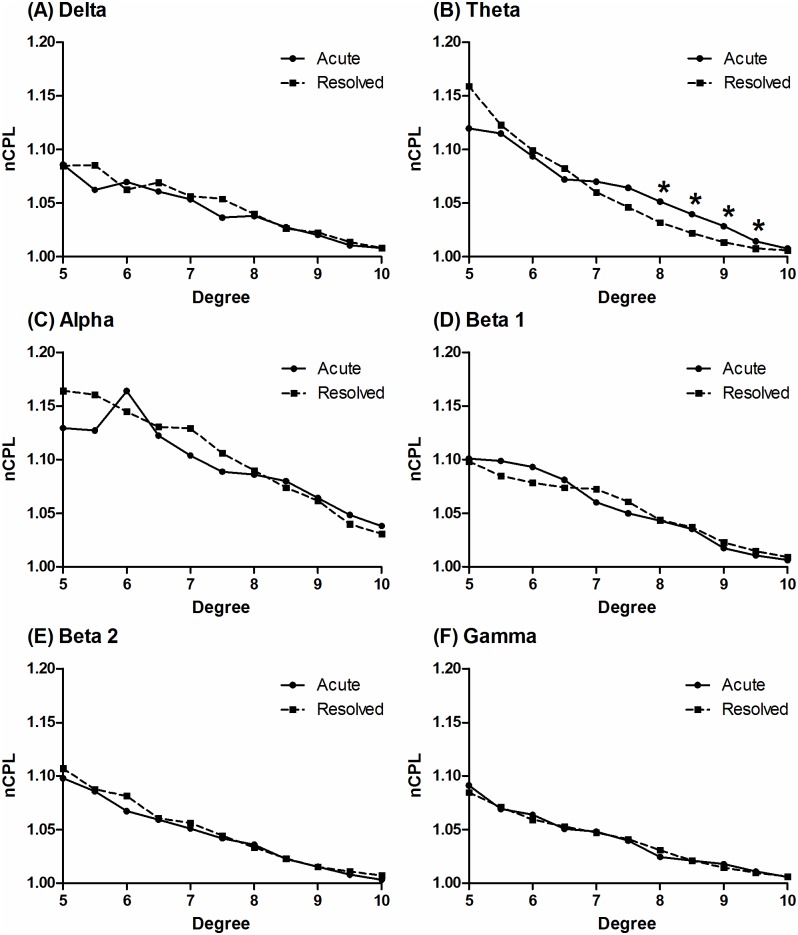
Comparison of the nCPL between the acute and resolved stages of TGA. The median nCPL values in the acute and resolved stage of TGA are presented with regard to the delta (A), theta (B), alpha (C), beta 1 (D), beta 2 (E) and gamma (F) frequency bands. The values of nCPL were computed as a function of the degree. Abbreviation: nCPL, normalized characterized path length; TGA, transient global amnesia. * *P* < 0.05, Wilcoxon signed-rank test.

**Fig 2:**
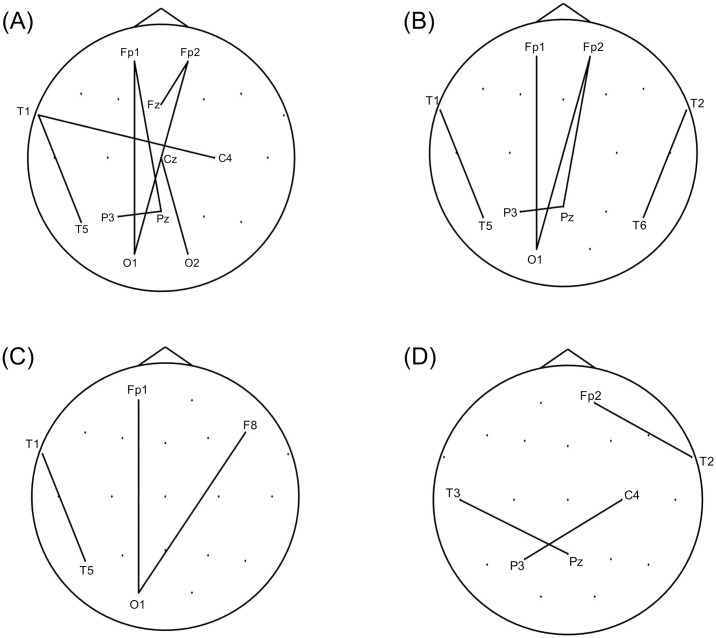
Edges lost during the acute stage of transient global amnesia when nCPL significantly increased. Edges were lost during the acute stage compared with the resolved stage for networks with mean degrees of 8 (A), 8.5 (B), 9 (C) and 9.5 (D) in the theta band when nCPL significantly increased. Most of the edges were between the anterior (e.g., Fp1, Fp2, T1 and T2) and posterior (e.g., T5, T6, Pz and O1) electrodes. Abbreviation: nCPL, normalized characterized path length.

**Fig 3:**
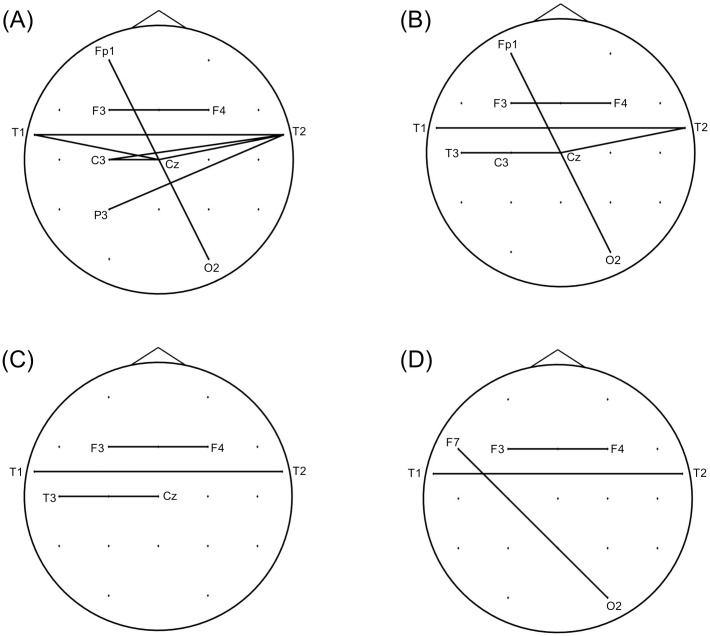
Edges developed during the acute stage of transient global amnesia when nCPL significantly increased. Edges were developed during the acute stage compared with the resolved stage for networks with mean degrees of 8 (A), 8.5 (B), 9 (C) and 9.5 (D) in the theta band when nCPL significantly increased. Most of the edges occurred between the left (e.g., F3 and T1) and right (e.g., F4 and T2) frontotemporal electrodes. Abbreviation: nCPL, normalized characterized path length.

The baseline characteristics of the study population are summarized in Table 1. In the theta frequency band, the nCPL of the acute stage was significantly increased compared with that of the resolved stage at mean degrees of 8 (*P* = 0.0273, Cohen’s *d* = 0.440), 8.5 (*P* = 0.0496, Cohen’s *d* = 0.416), 9 (*P* = 0.0355, Cohen’s *d* = 0.481) and 9.5 (*P* = 0.0250, Cohen’s *d* = 0.587; Fig 1). During the acute stage, lost edges for these networks mostly occurred between the anterior (e.g., Fp1, Fp2, T1 and T2) and posterior (e.g., T5, T6, Pz and O1) electrodes, whereas newly developed edges primarily occurred between the left (e.g., F3 and T1) and right (e.g., F4 and T2) frontotemporal electrodes (Figs 2 and 3). Furthermore, the Bonacich centrality of the Fp1 electrode was decreased at mean degrees of 8, 8.5, 9 and 9.5 during the acute stage. The Bonacich centrality of the Fp2 electrode was also decreased at mean degrees of 9 and 9.5 (S1 Table).

**Table 1:** Baseline characteristics of the study population.

**Age in years, mean (standard deviation)**	61.81 (8.65)
**Males**	6 (28.57%)
**Precipitating factor**	
**Physical stress**	4 (19.05%)
**Emotional stress**	7 (33.33%)
**Temperature change**	4 (19.05%)
**Severe pain**	1 (4.76%)
**Intercourse**	1 (4.76%)
**Associated symptom**	
**Headache**	2 (9.52%)
**Nausea**	3 (14.29%)
**Hypertension**	6 (28.57%)
**Diabetes**	1 (4.76%)
**Hyperlipidemia**	9 (42.86%)
**Hours from symptom onset to the initial DWI, median (interquartile range)**	7 (5–9)
**Laterality of diffusion-weighted imaging lesion**	
**Left**	8 (28.10%)
**Right**	9 (42.86%)
**Bilateral**	4 (19.05%)
**Location of diffusion-weighted imaging lesion**	
**Head**	6 (28.57%)
**Body**	11 (52.38%)
**Tail**	5 (23.81%)
**Days from symptom onset to the EEG recording during the resolved stage, median (interquartile range)**	147 (99–375)

Values are presented as numbers (%) unless otherwise indicated.

Also in the theta frequency band, the nCC of the acute stage was significantly decreased compared to that of the resolved stage at a mean degree of 5.5 (*P* = 0.0355, Cohen’s *d* = -0.527) (S2 Fig). Edges that were lost and developed during the acute stage for that network are presented in S3 Fig. The Bonacich centrality was not significantly different between the acute and resolved stage at a mean degree of 5.5 in the theta band (S1 Table). The effect sizes for the nCPL and nCC were medium, with Cohen’s *d* ranging from 0.416 to 0.587 [24]. For networks in the delta, alpha, beta1, beta 2 and gamma frequency bands, the nCPL and nCC did not significantly differ between the acute and resolved stages.

When the nCPL values from the patients with acute TGA were compared with those from the control subjects, there were no significant differences in any of the frequency bands. Additionally, the nCC values were significantly decreased in the patients with acute TGA compared to the control subjects in the theta frequency band at a mean degree of 7.5 (*P* = 0.0427, Cohen’s *d* = 0.5831) (S4 Fig).

## Discussion

Normally, spontaneous synchronization of the neural network at approximately 10 Hz regulates the fluctuation of the global arousal and consciousness states in a resting-state, eye-closed condition [27]. It was recently suggested that the spectral power of the alpha band in the parieto-occipital region is decreased during acute TGA, with a concomitant increase in the theta power associated with the dysfunction of the inhibitory neurons in the hippocampal CA1 field [17]. The results of our graph theoretical analysis of the resting-stage EEG data indicate that, although the theta power of the parieto-occipital region increased in a previous study [17], the network efficiency of the theta band was impaired during acute TGA. This result was manifested by the increased nCPL.

The theta rhythm has been strongly implicated in the mnemonic function of the hippocampus [28]. Hippocampal cell assemblies are activated in the theta cycle during the formation of episodic memory [29]. When the hippocampal theta rhythm was reduced after medial septal lesion, performance deteriorated on spatial and non-spatial memory tasks [30]. In addition, electrical stimulation of the Schaffer collateral-CA1 synapses at the theta frequency was optimized for the induction of hippocampal long-term potentiation compared with stimulation at shorter or longer intervals [31]. However, both the amount of theta rhythm and the functional connectivity in the theta band contribute to memory formation [32,33]. Theta coherence between the hippocampus and parahippocampal cortex [32] as well as across cortical regions [33] increases during memory formation.

In our study, lost edges mostly occurred between the anterior and posterior brain regions for networks in which the nCPL increased significantly during acute TGA. The Bonacich centrality of the anterior brain region (Fp1, Fp2) also decreased during this stage. A previous study reported that theta coherence between the frontal and posterior electrodes was enhanced during memory task performance [33]. We suggest that desynchronization in the theta band between the anterior and posterior brain regions might contribute to the episodic memory impairment that is associated with TGA. In contrast, the development of new edges between the left and right frontotemporal regions during acute TGA might be a manifestation of a compensatory process. The brain attempts to maximize performance in the face of damage by using structures or networks not typically engaged, a process known as cognitive reserve [34]. However, these connections might also represent a false-positive result. When we compared the network structures for the acute and resolved stages of TGA, we assumed that the two networks were equal in size. However, given that overall functional connectivity in the episodic memory network is reduced during the acute stage of TGA [6], the entire network size might be smaller during that time.

We also found that the nCC decreased in the theta band at a mean degree of 5.5. Given that the results were not corrected for multiple comparisons, differences at a single degree might be insufficient evidence to draw any conclusions. The relative sparing of the nCC suggests that the local connectedness of these brain networks might not be markedly disrupted following the hippocampal injury that occurs during acute TGA.

Although the nCC values for the patients with acute TGA were decreased compared to those of the control subjects in the theta band at a mean degree of 7.5, the nCPL values did not significantly different between these groups. Because the statistical power of a paired comparison could be greater than that of an independent comparison [35], it is possible that no significant differences would be detected between patients with acute TGA and control subjects, despite the differences in nCPL between acute and resolved TGA. The differences between the patients with acute TGA and the control subjects should be further investigated with larger samples in the future.

This study has limitations. First, the results were not corrected for multiple comparisons. When testing multiple hypotheses regarding the same issue, the individual *P* values of the tests might not be an appropriate guide for determining actual significance [36]. However, because adjustments for multiple tests increase the chance of type II errors and make the interpretation of findings dependent on the number of tests performed, simply describing the tests of significance that were performed is also a way to deal with multiple comparisons in exploratory studies [37]. Second, we used scalp EEG data collected from 21 electrodes according to the international 10–20 system, which might not be optimal for graph theoretical analysis. Using a great number of electrodes provides higher spatial resolution and enables the modeling of the brain network at the cortical level. However, because EEG data are usually acquired according to the 10–20 system in the clinical setting, many clinical studies use these EEG data despite their limitations for research purposes [38].

In conclusion, we demonstrated that network efficiency in the theta band is impaired during the acute stage of TGA using graph theoretical analysis. The desynchronization between the anterior and posterior brain regions might contribute to the clinical manifestation of TGA.

## Supporting Information

S1 Fig Examples of hippocampal lesions on diffusion-weighted imaging in patients with transient global amnesia. Punctuate hyperintense lesions in the head (A), body (B) and tail (C) of the hippocampus are indicated with white arrows on axial diffusion-weighted imaging. Modified from Park et al. [5].
(TIF)

S2 Fig Comparison of the nCC between the acute and resolved stages of TGA. The median nCC values during the acute and resolved stages of TGA are presented with regard to the delta (A), theta (B), alpha (C), beta 1 (D), beta 2 (E) and gamma (F) frequency bands. The values of nCC were computed as a function of the degree. Abbreviation: nCC, normalized clustering coefficient; TGA, transient global amnesia. * *P* < 0.05, Wilcoxon signed-rank test.
(TIF)

S3 Fig Edges lost and developed during the acute stage of transient global amnesia when nCC significantly decreased. Edges were lost (A) and developed (B) during the acute stage compared with the resolved stage for a network at a mean degree of 5.5 in the theta band when nCC significantly decreased. A marked difference was not observed between the lost and developed edges. Abbreviation: nCC, normalized clustering coefficient.
(TIF)

S4 Fig Comparison of the nCC between patients with acute TGA and control subjects. The median nCC values for patients with acute TGA patients and control subjects are presented with regard to the delta (A), theta (B), alpha (C), beta 1 (D), beta 2 (E) and gamma (F) frequency bands. The values of nCC were computed as a function of the degree. Abbreviation: nCC, normalized clustering coefficient; TGA, transient global amnesia. * *P* < 0.05, Wilcoxon signed-rank test.
(TIF)

S1 Table Comparison of the Bonacich centrality of each electrode between the acute and resolved stages of transient global amnesia in the theta frequency band at mean degrees of 5.5, 8, 8.5, 9 and 9.5. Abbreviations: IQR, interquartile range. *The P values were obtained by Wilcoxon signed-rank test.
(PDF)
