# Supplementary figures and images for: Transient Global Amnesia Deteriorates the Network Efficiency of the Theta Band
Source: PLoS One. 2016 Oct 14;11(10):e0164884. doi: 10.1371/journal.pone.0164884 (PMC5065218; doi:10.1371/journal.pone.0164884)

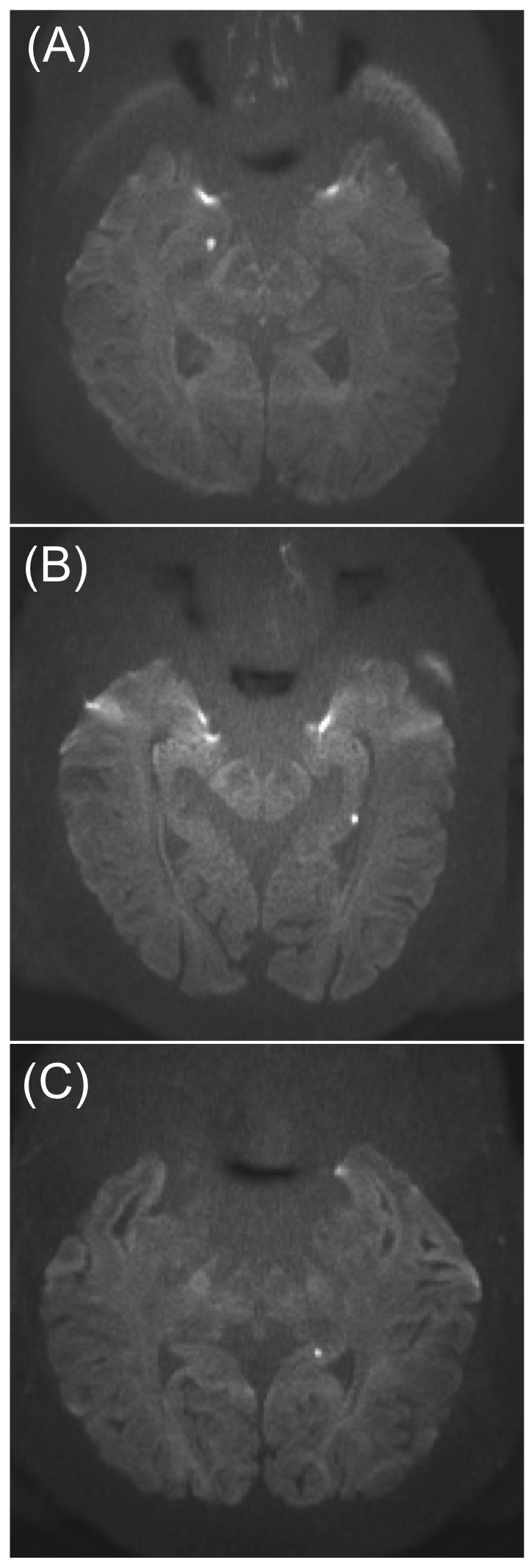

Supplement: S1 Fig — Punctuate hyperintense lesions in the head (A), body (B) and tail (C) of the hippocampus are indicated with white arrows on axial diffusion-weighted imaging. Modified from Park et al. [5]. (TIF) [file pone.0164884.s001.tif]

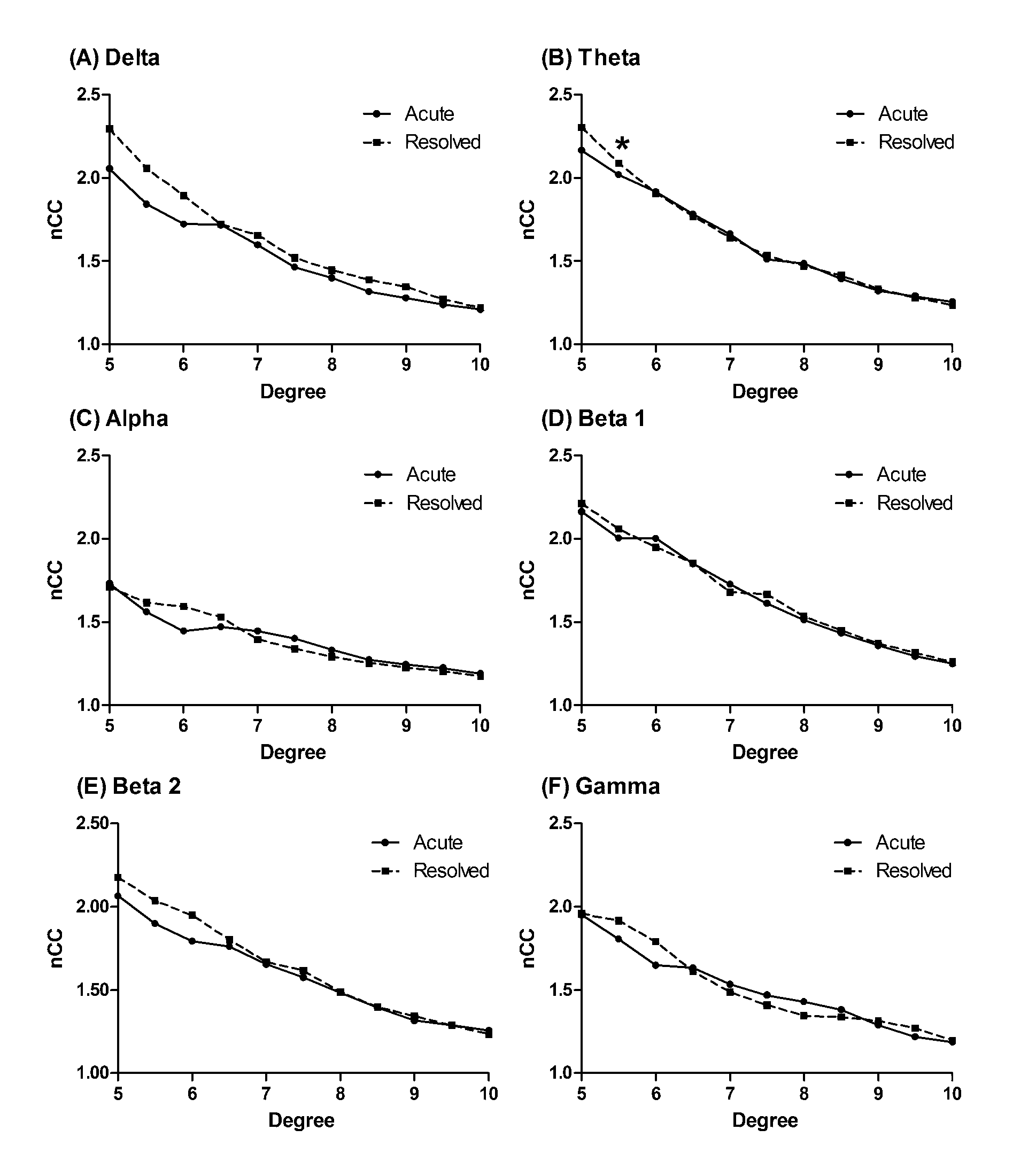

Supplement: S2 Fig — The median nCC values during the acute and resolved stages of TGA are presented with regard to the delta (A), theta (B), alpha (C), beta 1 (D), beta 2 (E) and gamma (F) frequency bands. The values of nCC were computed as a function of the degree. Abbreviation: nCC, normalized clustering coefficient; TGA, transient global amnesia. * P < 0.05, Wilcoxon signed-rank test. (TIF) [file pone.0164884.s002.tif]

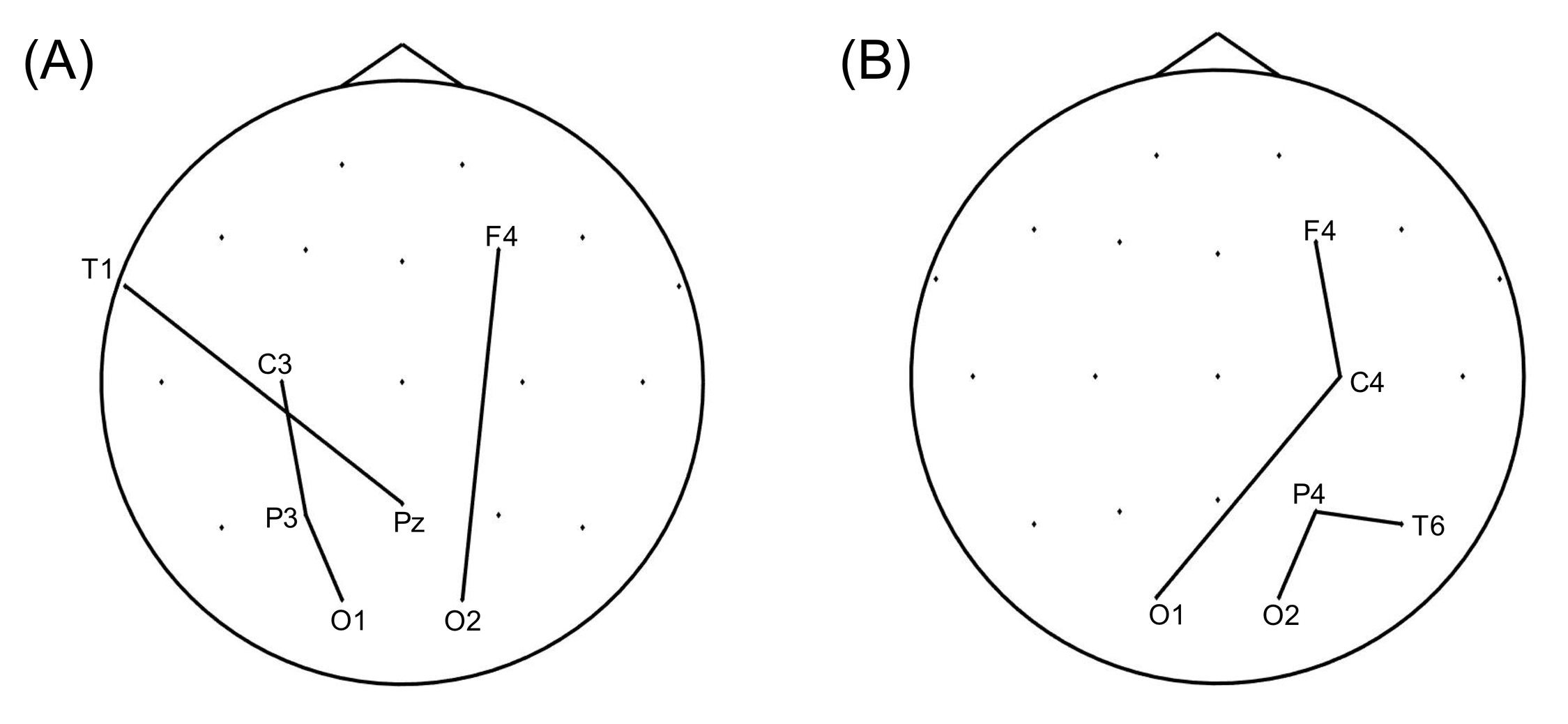

Supplement: S3 Fig — Edges were lost (A) and developed (B) during the acute stage compared with the resolved stage for a network at a mean degree of 5.5 in the theta band when nCC significantly decreased. A marked difference was not observed between the lost and developed edges. Abbreviation: nCC, normalized clustering coefficient. (TIF) [file pone.0164884.s003.tif]

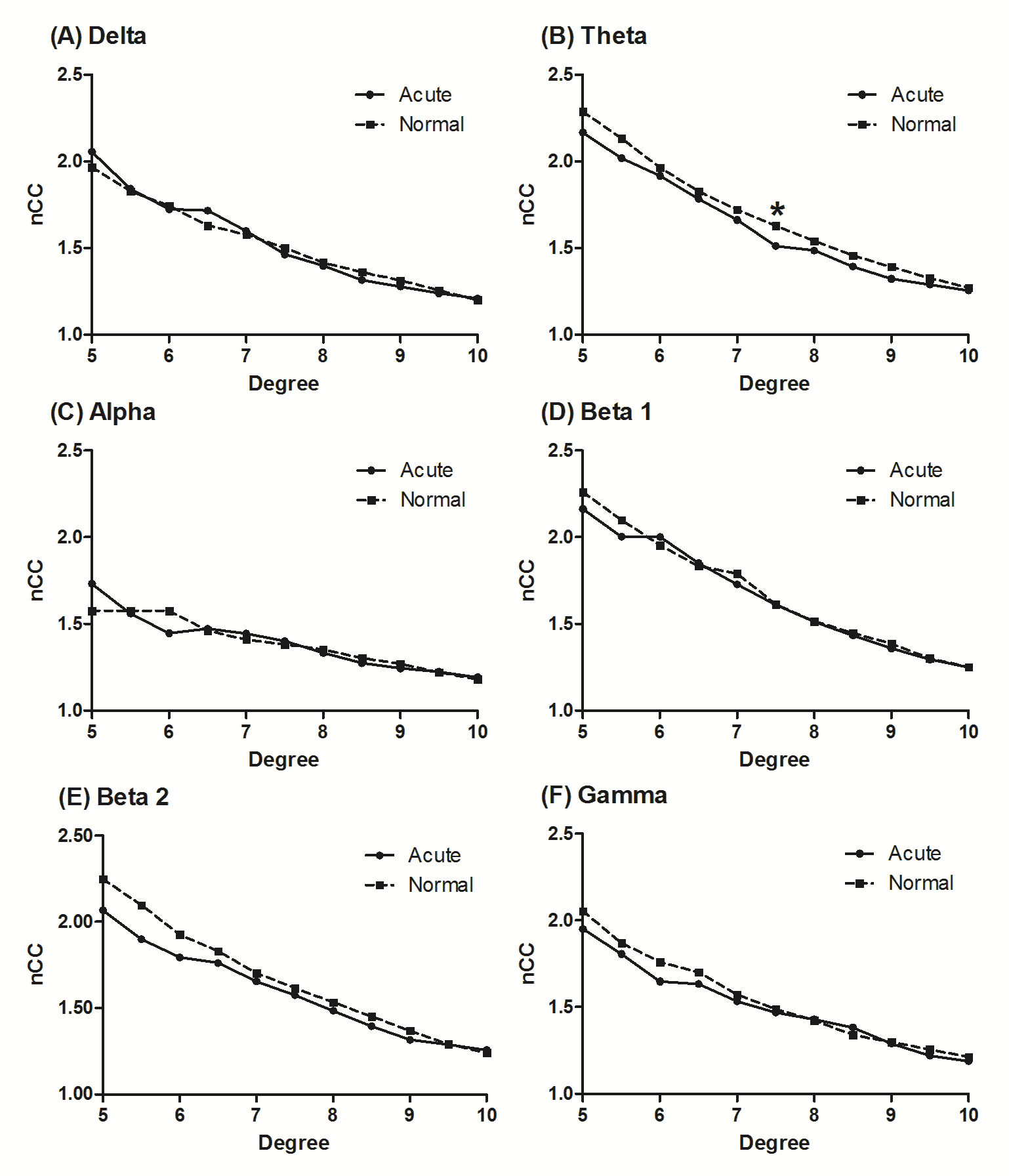

Supplement: S4 Fig — The median nCC values for patients with acute TGA patients and control subjects are presented with regard to the delta (A), theta (B), alpha (C), beta 1 (D), beta 2 (E) and gamma (F) frequency bands. The values of nCC were computed as a function of the degree. Abbreviation: nCC, normalized clustering coefficient; TGA, transient global amnesia. * P < 0.05, Wilcoxon signed-rank test. (TIF) [file pone.0164884.s004.tif]
