# Supplementary material for: Transient Global Amnesia Deteriorates the Network Efficiency of the Theta Band
Source: PLoS One. 2016 Oct 14;11(10):e0164884. doi: 10.1371/journal.pone.0164884 (PMC5065218; doi:10.1371/journal.pone.0164884)
Supplement: S1 Table — Abbreviations: IQR, interquartile range. *The P values were obtained by Wilcoxon signed-rank test. (PDF) [file pone.0164884.s005.pdf]

S1 Table. Comparison of the Bonacich centrality of each electrode between the acute and resolved stages of transient global amnesia in the theta frequency band at mean degrees of 5.5, 8, 8.5, 9 and 9.5.

Mean Degree	EEG electrode	Median (IQR) for the acute stage	Median (IQR) for the resolved stage	*P* value*
5.5	Fp1	0.1313 (0.0677-0.2061)	0.1510 (0.0888-0.2491)	0.2443
	F3	0.3064 (0.1503-0.3420)	0.2529 (0.1414-0.3257)	0.4549
	C3	0.1674 (0.0516-0.2757)	0.1485 (0.0510-0.2023)	0.3392
	P3	0.1549 (0.0806-0.3024)	0.2240 (0.0442-0.3024)	0.5202
	Fp2	0.1123 (0.0672-0.2516)	0.1512 (0.0995-0.2562)	0.3945
	F4	0.2795 (0.1628-0.3451)	0.2858 (0.1955-0.3481)	0.6389
	C4	0.0960 (0.0583-0.2235)	0.1482 (0.0566-0.2543)	0.4761
	P4	0.1323 (0.0766-0.2796)	0.1505 (0.0794-0.2173)	0.9308
	F7	0.1214 (0.0459-0.2128)	0.1674 (0.1149-0.1896)	0.5663
	T3	0.1328 (0.0624-0.2240)	0.1530 (0.0544-0.2257)	0.5430
	T5	0.2106 (0.0836-0.3474)	0.2101 (0.1251-0.3514)	0.4140
	O1	0.2580 (0.1497-0.3226)	0.2789 (0.2511-0.3472)	0.2305
	F8	0.1501 (0.0684-0.2370)	0.1410 (0.0746-0.2845)	0.9032
	T4	0.1295 (0.0585-0.2524)	0.0962 (0.0550-0.2268)	0.7413
	T6	0.2194 (0.1349-0.3080)	0.2075 (0.1113-0.2905)	0.1924
	O2	0.2528 (0.1210-0.3187)	0.2786 (0.1923-0.3302)	0.0582
	T1	0.1008 (0.0320-0.1576)	0.0991 (0.0533-0.1794)	0.4549
	T2	0.0781 (0.0430-0.2086)	0.0809 (0.0254-0.1874)	0.8757
	Fz	0.3193 (0.1625-0.4046)	0.3245 (0.2550-0.3596)	0.6143
	Cz	0.1673 (0.0645-0.2828)	0.1629 (0.0576-0.2893)	0.8213
	Pz	0.0922 (0.0249-0.1912)	0.1107 (0.0467-0.2159)	0.8228
8	Fp1	0.1828 (0.1000-0.2685)	0.2227 (0.1668-0.2802)	0.0143
	F3	0.2498 (0.1999-0.3074)	0.2614 (0.2221-0.3104)	0.3392
	C3	0.1873 (0.1186-0.2481)	0.1764 (0.1286-0.2446)	0.9308
	P3	0.1992 (0.1682-0.2581)	0.2018 (0.1556-0.2716)	0.3570
	Fp2	0.2000 (0.1354-0.2448)	0.1980 (0.1327-0.2791)	0.3754
	F4	0.2567 (0.2288-0.3125)	0.2607 (0.2289-0.2990)	0.9032
	C4	0.1894 (0.1413-0.2221)	0.1792 (0.1375-0.2252)	0.5663
	P4	0.2197 (0.1441-0.2472)	0.2221 (0.1682-0.2695)	0.7151
	F7	0.1752 (0.1131-0.2621)	0.1883 (0.1278-0.2323)	0.8213
	T3	0.2016 (0.1138-0.2373)	0.1603 (0.1074-0.2361)	0.2443
	T5	0.1967 (0.1568-0.2738)	0.2251 (0.1648-0.2641)	0.7943
	O1	0.2427 (0.2051-0.2986)	0.2671 (0.2151-0.3065)	0.5663
	F8	0.1859 (0.1558-0.2221)	0.1628 (0.1099-0.2346)	0.4140
	T4	0.1469 (0.0948-0.1944)	0.1580 (0.1191-0.1661)	0.7943
	T6	0.2102 (0.1815-0.2718)	0.2029 (0.1696-0.2455)	0.5430
	O2	0.2632 (0.1910-0.3116)	0.2461 (0.1919-0.3041)	0.6143
	T1	0.1596 (0.1238-0.2151)	0.1467 (0.0905-0.2104)	0.3392
	T2	0.1495 (0.0765-0.1997)	0.1237 (0.0886-0.1990)	0.5901
	Fz	0.2845 (0.2360-0.3098)	0.2805 (0.2554-0.3054)	0.7151

	Cz	0.2155 (0.1511-0.3084)	0.2456 (0.1521-0.2989)	0.9308
	Pz	0.1810 (0.0911-0.2795)	0.1952 (0.1351-0.2400)	0.9584
8.5	Fp1	0.2049 (0.0951-0.2574)	0.2343 (0.1786-0.2626)	0.0298
	F3	0.2483 (0.2253-0.3189)	0.2521 (0.2179-0.2960)	0.9308
	C3	0.1938 (0.1151-0.2327)	0.1912 (0.1269-0.2547)	0.9861
	P3	0.1862 (0.1716-0.2596)	0.2009 (0.1580-0.2725)	0.5430
	Fp2	0.1977 (0.1404-0.2350)	0.2083 (0.1391-0.2804)	0.3754
	F4	0.2672 (0.2314-0.3075)	0.2602 (0.2220-0.3018)	0.9032
	C4	0.1896 (0.1563-0.2196)	0.2047 (0.1518-0.2345)	0.7151
	P4	0.2091 (0.1599-0.2521)	0.2287 (0.1788-0.2635)	0.5663
	F7	0.1940 (0.1408-0.2470)	0.1987 (0.1289-0.2401)	0.9584
	T3	0.1914 (0.1084-0.2400)	0.1577 (0.1086-0.2189)	0.2046
	T5	0.1985 (0.1595-0.2541)	0.2236 (0.1745-0.2567)	0.9308
	O1	0.2431 (0.2093-0.3047)	0.2582 (0.2090-0.3069)	0.5430
	F8	0.1945 (0.1550-0.2185)	0.1609 (0.1265-0.2195)	0.2046
	T4	0.1465 (0.0901-0.1841)	0.1448 (0.1287-0.1665)	0.8484
	T6	0.2147 (0.1803-0.2661)	0.2179 (0.1820-0.2497)	0.7943
	O2	0.2653 (0.1805-0.2942)	0.2520 (0.1870-0.2963)	0.6639
	T1	0.1780 (0.1243-0.2087)	0.1471 (0.1012-0.2267)	0.7943
	T2	0.1476 (0.0833-0.2139)	0.1521 (0.0806-0.1826)	0.2736
	Fz	0.2727 (0.2210-0.3104)	0.2810 (0.2563-0.3066)	0.5202
	Cz	0.2294 (0.1598-0.3038)	0.2330 (0.1514-0.2854)	0.9308
	Pz	0.1924 (0.1000-0.2683)	0.1817 (0.1349-0.2479)	0.9861
9	Fp1	0.2104 (0.1313-0.2457)	0.2468 (0.1881-0.2713)	0.0386
	F3	0.2502 (0.2267-0.3015)	0.2536 (0.2160-0.2924)	0.4979
	C3	0.1978 (0.1388-0.2297)	0.1839 (0.1518-0.2471)	0.7943
	P3	0.2082 (0.1611-0.2657)	0.2154 (0.1597-0.2793)	0.3754
	Fp2	0.1869 (0.1301-0.2470)	0.2210 (0.1576-0.2677)	0.0457
	F4	0.2651 (0.2517-0.3003)	0.2542 (0.2218-0.2913)	0.3945
	C4	0.1891 (0.1603-0.2148)	0.2120 (0.1482-0.2412)	0.6894
	P4	0.2078 (0.1613-0.2557)	0.2120 (0.1912-0.2594)	0.4342
	F7	0.1804 (0.1386-0.2562)	0.1949 (0.1298-0.2287)	0.8484
	T3	0.2019 (0.1063-0.2375)	0.1628 (0.1093-0.2130)	0.1396
	T5	0.2130 (0.1667-0.2530)	0.2113 (0.1830-0.2548)	0.7677
	O1	0.2500 (0.2232-0.2935)	0.2539 (0.2147-0.2983)	0.9308
	F8	0.1833 (0.1522-0.2201)	0.1766 (0.1255-0.2157)	0.4342
	T4	0.1456 (0.0998-0.2019)	0.1519 (0.1287-0.1700)	0.9861
	T6	0.2094 (0.1831-0.2623)	0.2182 (0.1821-0.2513)	0.8757
	O2	0.2711 (0.1828-0.2850)	0.2409 (0.1930-0.3030)	0.4979
	T1	0.1873 (0.1265-0.2208)	0.1487 (0.1124-0.2066)	0.2172
	T2	0.1421 (0.0931-0.2073)	0.1545 (0.0878-0.1750)	0.1396
	Fz	0.2658 (0.2174-0.2967)	0.2796 (0.2537-0.3059)	0.4140
	Cz	0.2341 (0.1816-0.3022)	0.2165 (0.1588-0.2658)	0.3945
	Pz	0.2018 (0.1150-0.2631)	0.1793 (0.1583-0.2657)	0.6143
9.5	Fp1	0.2113 (0.1529-0.2474)	0.2364 (0.1972-0.2778)	0.0208

	F3	0.2691 (0.2119-0.2834)	0.2569 (0.2171-0.2820)	0.7677
	C3	0.1962 (0.1486-0.2301)	0.2137 (0.1580-0.2261)	0.7943
	P3	0.2088 (0.1617-0.2533)	0.2383 (0.1609-0.2694)	0.1305
	Fp2	0.1987 (0.1443-0.2547)	0.2209 (0.1743-0.2551)	0.0496
	F4	0.2661 (0.2430-0.2915)	0.2552 (0.2233-0.2770)	0.1138
	C4	0.1923 (0.1590-0.2199)	0.2103 (0.1600-0.2451)	0.4140
	P4	0.2200 (0.1673-0.2591)	0.2177 (0.1985-0.2477)	0.6894
	F7	0.1727 (0.1503-0.2478)	0.2010 (0.1404-0.2305)	0.9032
	T3	0.1959 (0.1101-0.2228)	0.1615 (0.1273-0.2078)	0.2736
	T5	0.2159 (0.1780-0.2662)	0.2181 (0.1842-0.2436)	0.4549
	O1	0.2542 (0.2167-0.2958)	0.2619 (0.2152-0.2815)	0.8213
	F8	0.1978 (0.1506-0.2312)	0.1870 (0.1307-0.2135)	0.6894
	T4	0.1567 (0.1072-0.2086)	0.1603 (0.1313-0.1840)	0.9861
	T6	0.2107 (0.1807-0.2490)	0.2191 (0.1831-0.2401)	0.9861
	O2	0.2538 (0.1922-0.2809)	0.2281 (0.2035-0.2845)	0.9032
	T1	0.1761 (0.1308-0.2192)	0.1529 (0.1178-0.1956)	0.1924
	T2	0.1350 (0.0942-0.2082)	0.1520 (0.1039-0.1811)	0.2305
	Fz	0.2629 (0.2222-0.2901)	0.2753 (0.2417-0.2988)	0.4761
	Cz	0.2453 (0.1856-0.3004)	0.2340 (0.1437-0.2781)	0.3754
	Pz	0.2217 (0.1334-0.2760)	0.1972 (0.1617-0.2670)	0.5202

Abbreviation: IQR, interquartile range.

*The *P* values were obtained by Wilcoxon signed-rank test.
